# Health Risk Assessment of Antibiotic Residues in Normally Slaughtered, Emergency-Slaughtered, and Imported Beef in Egypt

**DOI:** 10.3390/antibiotics15090932

**Published:** 2026-09-20

**Authors:** Mohammed Moheb Abdelhameed, Hend Ali Elshebrawy, Kálmán Imre, Adriana Morar, Samir Mohammed Abd-Elghany, Khalid Ibrahim Sallam

**Affiliations:** 1 Department of Food Hygiene, Safety, and Technology, Faculty of Veterinary Medicine, Mansoura University, Mansoura 35516, Egypt; mohammed.moheb86@std.mans.edu.eg (M.M.A.); hend_ali85@mans.edu.eg (H.A.E.); drsamir@mans.edu.eg (S.M.A.-E.); 2 Faculty of Veterinary Medicine, University of Life Sciences “King Mihai I” from Timisoara, 300645 Timisoara, Romania; adrianamorar@usvt.ro

**Keywords:** beef, antibiotic residues, oxytetracycline, ciprofloxacin, sulphadimidine, HPLC, health hazards

## Abstract

**Background/Objectives:** Despite the widespread use of antibiotics in animal husbandry, limited data are available on the presence of antibiotic residues in beef consumed in Egypt. Therefore, the current study aimed to determine ciprofloxacin, oxytetracycline, and sulphadimidine residues in beef marketed in Ismailia, Egypt. **Methods:** A total of 300 beef samples (100 each of normally slaughtered carcasses, emergency-slaughtered carcasses, and imported frozen beef cuts) were collected and analyzed for ciprofloxacin, oxytetracycline, and sulphadimidine residues using high-performance liquid chromatography (HPLC). Dietary exposure and potential health risks were assessed using the hazard quotient (*HQ*). **Results:** The findings demonstrated that 27.7% (83/300), 37.0% (111/300), and 41.7% (125/300) of beef samples examined were contaminated with ciprofloxacin, oxytetracycline, and sulphadimidine residues, respectively. The mean concentrations (µg/kg) for ciprofloxacin, oxytetracycline, and sulphadimidine were 136.7, 245.5, and 209.7 in normally slaughtered beef; 260.2, 378.6, and 301.4 in emergency-slaughtered beef; and 242.8, 261.4, and 115.7 in imported frozen beef, respectively. Notably, 13%, 26%, and 22% of the samples analyzed exceeded the maximum residue limits (MRLs) established by the European Union and the Codex Alimentarius Commission for ciprofloxacin, oxytetracycline, and sulphadimidine, respectively. Emergency-slaughtered beef exhibited higher mean concentrations of antibiotic residues and a greater proportion of samples exceeding the MRLs. The *HQ* values of each antibiotic residue were higher in schoolchildren than in adults, with emergency-slaughtered beef samples exhibiting the highest *HQ* values for all three antibiotics. **Conclusions:** The detection of antibiotic residues, including concentrations exceeding established MRLs, particularly in emergency-slaughtered beef, highlights the crucial importance of complying with antibiotic withdrawal periods and rationalizing their use in veterinary medicine to protect public health.

## 1. Introduction

Red meat is rich in high-biological-value protein, saturated and unsaturated fats, and vital micronutrients, including iron, zinc, selenium, and vitamin B12, which are essential for human health [1]. Cattle and buffalo represent the primary sources of ruminant meat in Egypt. According to recent national statistics from the Central Agency for Public Mobilization and Statistics (CAPMAS), the average per capita consumption of red meat was 8.0 kg/year [2]. With a national self-sufficiency rate of 55.4%, a substantial supply deficit remains, which is bridged through imported frozen beef and live cattle to meet consumer demand [2]. Red meat production in Egypt exhibited a significant downward trend during 2003–2022, decreasing by approximately 17.3 thousand tons annually (≈2.1% of the average production of 842 thousand tons), a statistically significant reduction. In contrast, red meat consumption showed a slight, non-significant downward trend of about 6.1 thousand tons per year (≈0.5% of the average consumption) [3]. Nevertheless, a persistent production–consumption gap remains, resulting in continued reliance on imports [3]. Bovine meat consumption in Egypt depends mainly on native fresh beef, imported live cattle from Sudan and Brazil for immediate slaughter, frozen beef from Brazil, and frozen water buffalo meat from India [4]. Nonetheless, Egyptian consumers prefer freshly slaughtered native beef to frozen beef owing to cultural habits [4].

Animals slaughtered under normal conditions must be healthy, clean, adequately rested before slaughter, and subjected to ante-mortem inspection by a veterinarian and, after slaughter, to rigorous post-mortem inspection of the carcasses of slaughtered cattle, as part of the regulations on the so-called hygiene package, and then marked as fit for human consumption without any precautions. On the other hand, food animals may be slaughtered for human consumption without ante-mortem inspection in emergency cases, such as milk fever, mastitis, dystocia, fractures, trauma, rectal prolapse, and other infectious diseases, and this is called on-farm emergency slaughter [5,6]. According to the resolution of the Minister of Agriculture and Land Reclamation No. 517 of 1986 regarding animal slaughter and meat trade in Egypt, in case of emergency slaughter, the owner of the slaughtered animal must go to the nearest police station to report the case, and he must immediately deliver it to the nearest abattoir, encompassing the whole carcass with its viscera intact without separating any part of it [7]. In some situations, on-farm emergency slaughter is a golden opportunity for farmers to recover part of the economic worth of an animal that is unsuitable for transport to slaughterhouses [7].

In livestock production, antibiotics are widely administered for prophylactic, therapeutic, and growth promotion purposes [8,9]. The presence of antibiotic residues or their metabolites in foods of animal origin represents a potential public health concern [10]. Such antibiotics could also exceed the maximum residue limits (MRLs), contributing to the development of antibiotic-resistant microorganisms, which make certain human infections difficult or even impossible to treat [11]. The occurrence of antibiotic residues in food commodities can be attributed to multiple factors, including non-compliance with withdrawal periods—the mandatory waiting time post-treatment to ensure residues drop below safe levels—often due to farmers’ lack of awareness or economic pressure to minimize losses [12]. Additional contributing circumstances include improper drug dosage, extra-label administration, emergency slaughter of actively treated animals, lack of farm treatment records, and insufficient regulatory oversight or veterinary supervision [11].

Global antibiotic consumption in livestock (cattle, sheep, chickens, and pigs) reached 99,502 tons in 2020 and is expected to increase by 8% by 2030 [13]. More recent estimates also indicate a 16.3% increase in global antibiotic consumption between 2016 and 2023, with the most pronounced increases occurring in upper-middle- and lower-middle-income countries [14]. Ciprofloxacin, enrofloxacin, oxytetracycline, chlortetracycline, sulfonamides, and nitrofurans are among the antibiotics commonly used in cattle [15]. Ciprofloxacin is used to treat several bacterial infections [12,16], whereas oxytetracycline is frequently used for the prevention and treatment of diseases such as mastitis, metritis, and bronchopneumonia [11]. Sulphadimidine is a synthetic sulfonamide used to treat bacterial and parasitic diseases and has also been used as a feed additive [17,18,19]. Misuse or overuse of these antibiotics and failure to observe withdrawal periods may result in the occurrence of their residues in meat and meat products, representing a potential public health concern [20].

To protect consumers, international and national authorities have established acceptable daily intake (*ADI*) values and MRLs for veterinary drug residues in foods [11,20]. The MRL for ciprofloxacin set by the European Union is 100 µg/kg [21], whereas the MRLs established by the Codex Alimentarius Commission for oxytetracycline and sulphadimidine are 200 and 100 µg/kg, respectively [22]. Antibiotic residues can be detected using screening and confirmatory analytical methods [23]. Among the available quantitative methods, high-performance liquid chromatography (HPLC) is widely used because of its high sensitivity and specificity [24,25,26,27,28,29,30,31,32,33,34].

Continuous dietary exposure to antibiotic residues may contribute to adverse health effects and the development of antibiotic-resistant bacteria [20]. Estimated daily intake (*EDI*) and hazard quotient (*HQ*) are commonly used to characterize dietary exposure and potential health risks [26,35,36,37,38]. Previous studies have assessed the health risks associated with antibiotic residues in sheep muscle, liver, and kidney in Kuwait [11] and in chicken meat [20,37]. However, information on the occurrence and health risk assessment of ciprofloxacin, oxytetracycline, and sulphadimidine residues in different categories of beef, particularly normally slaughtered beef, emergency-slaughtered beef, and imported frozen beef, remains limited in Egypt. Therefore, the present study aimed to quantify these antibiotic residues in beef marketed in Ismailia Governorate, Egypt, using HPLC and to assess the potential human health risks associated with their dietary exposure.

## 2. Results and Discussion

### 2.1. Method Validation for HPLC Quantification of Antibiotic Residues in Beef

HPLC analysis was used for the quantitative determination of antibiotic residues in the examined beef samples. The calibration curves for ciprofloxacin, oxytetracycline, and sulphadimidine showed good linearity and are shown in the supplementary figure (Figure S1A–C).

The chromatogram of the blank sample is shown in Figure S1D, while the chromatograms of the antibiotic standards and extracted meat samples are demonstrated in Figure S2A–F. No interference was observed at the retention times of ciprofloxacin (6.36 ± 0.03), oxytetracycline (6.06 ± 0.04), and sulphadimidine (4.85 ± 0.02), confirming the method’s selectivity and specificity.

### 2.2. Prevalence of Antibiotic Residues in Beef Samples

For the three antibiotics analyzed in the current study, 64% (64/100), 80% (80/100), and 63% (63/100) of the normally slaughtered beef, emergency-slaughtered beef, and imported frozen beef samples tested, respectively, were contaminated with antibiotic residues by HPLC analysis. The high prevalence of antibiotic residues could be related to the indiscriminate use of antibiotics in the veterinary sector, failure to adhere to withdrawal periods, and the emergency slaughter of sick animals [12,20], which constitute a tremendous challenge for the food industry to ensure meat safety.

### 2.3. Prevalence and Concentration of Ciprofloxacin Residues (μg/kg) in Beef Samples

Overall, 27.7% (83/300) of beef samples investigated in the current study were positive for ciprofloxacin. In this context, ciprofloxacin residues were found in 50% (40/80) of beef samples tested in Nigeria [39]. A higher prevalence of 100% (41/41) was detected for ciprofloxacin residues by ELISA analysis in beef samples examined in Tehran, Iran [40]. Likewise, ELISA analysis indicated the presence of ciprofloxacin residues in 93% (14/15) of beef muscle examined in South Africa, whereas HPLC did not detect any ciprofloxacin residues in the same samples [27]. In contrast, ciprofloxacin was not found in beef samples analyzed in Ghana [41]. Ciprofloxacin residues in beef samples may result from neglect of the withdrawal period or the metabolism of ciprofloxacin in the liver, considering its half-life ranges between 2 and 6 h [27].

**Figure 1:**
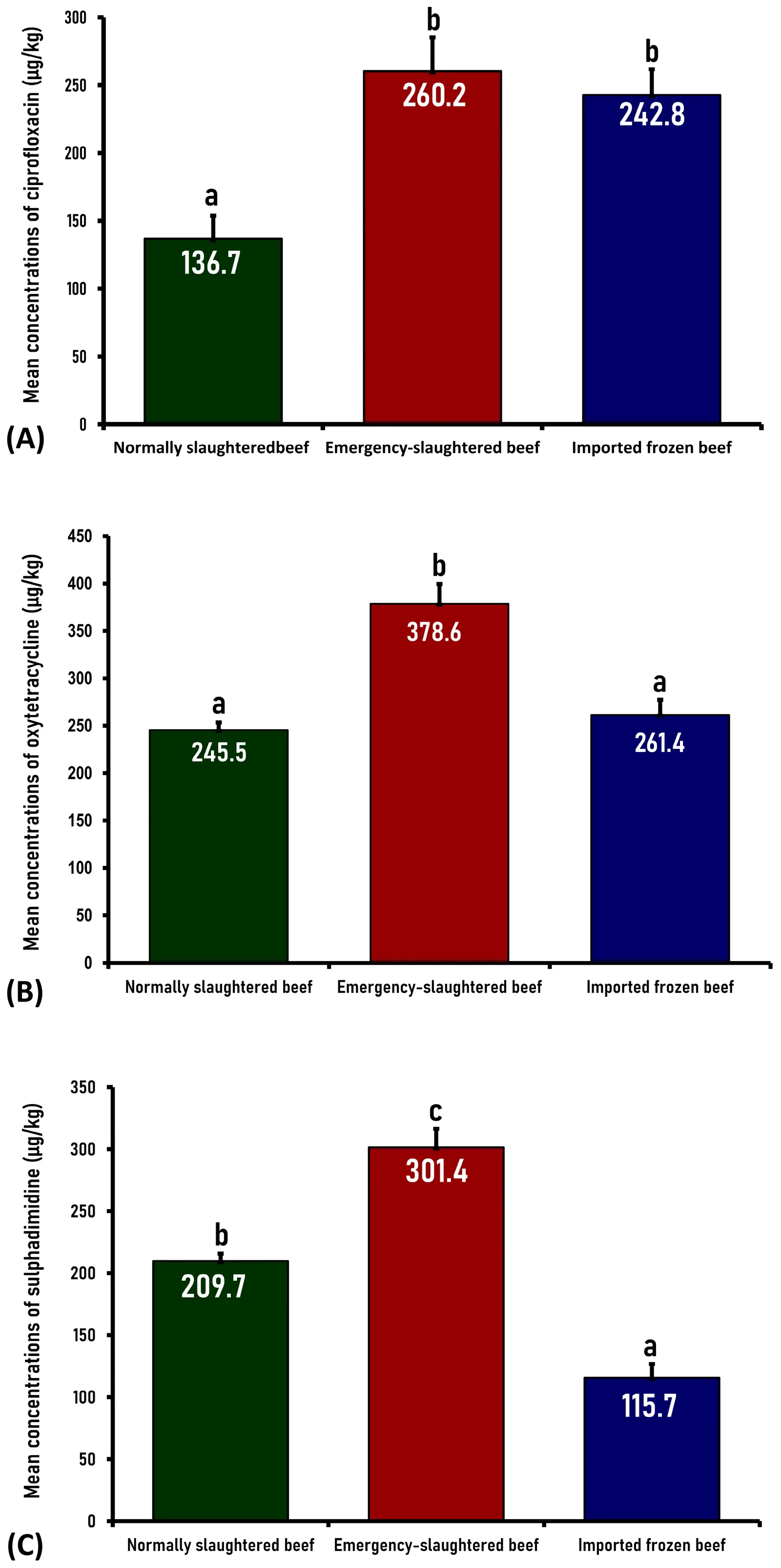
Mean ± SE concentration (µg/kg) of ciprofloxacin (**A**), oxytetracycline (**B**), and sulphadimidine (**C**) in normally slaughtered beef carcasses, emergency-slaughtered beef carcasses, and imported frozen beef cuts. Error bars represent the standard error (SE). Different lowercase letters (a, b, and c) above the columns indicate statistically significant differences among the beef categories (*p* < 0.05).

A much higher prevalence rate was reported by Ramatla et al. [27] in South Africa, who detected ciprofloxacin residues in 93% (14/15) of beef muscles tested, with mean ± SD values of 110.3 ± 9.4 µg/kg. Furthermore, 50% (40/80) of beef samples tested in Nigeria were positive for ciprofloxacin, with a higher mean value of 231.08 µg/kg [39]. Conversely, a much lower mean value of 8.3 µg/kg was determined for beef samples tested in Tehran, Iran [40]. At the level of beef categories tested in the current study, ciprofloxacin residues were detected in 26% (26/100), 39% (39/100), and 18% (18/100) of normally slaughtered beef, emergency-slaughtered beef, and imported frozen beef samples tested, respectively (Table 1). The concentrations of ciprofloxacin in normally slaughtered beef, emergency-slaughtered beef, and imported frozen beef samples examined ranged from 0.58 to 820, 5 to 1205, and 5.77 to 968 µg/kg, with mean ± SE values of 136.7 ± 17, 260.2 ± 25, and 242.8 ± 19 µg/kg, respectively (Figure 1A). Normally slaughtered meat exhibited significantly lower levels of ciprofloxacin (*p* < 0.05) than emergency-slaughtered beef and imported frozen beef (Figure 1A).

### 2.4. Prevalence and Concentration of Oxytetracycline Residues (μg/kg) in Beef Samples

Broad-spectrum antibiotics such as oxytetracycline and tetracycline are widely used in veterinary medicine to prevent and treat many livestock diseases. Tetracycline has a relatively short half-life of 7 to 10 h, with approximately 60% of the administered dose excreted unchanged in the urine [27].

The detection of oxytetracycline residues in 37% (111/300) of the examined beef samples indicates relatively frequent exposure of beef to this antimicrobial. The prevalence was higher in imported frozen beef (43%, 43/100) and emergency-slaughtered beef (38%, 38/100) than in normally slaughtered beef (30%, 30/100). Similarly, Nchima et al. [42] indicated that 34.4% (77/224) of beef samples examined in Zambia were positive for oxytetracycline. Additionally, around 48% of Somali goat samples collected from the Muscat Municipality Slaughterhouse in Oman contained oxytetracycline [43]. A higher detection frequency of tetracycline residues (75.6%, 31/41) was found in beef samples examined in Tehran, Iran [40]. Likewise, Bedada et al. [44] detected oxytetracycline residues in 71.3% (274/384) of cattle muscle samples collected from Addis Ababa, Debre Zeit, and Nazareth slaughterhouses in central Ethiopia. ELISA analysis indicated the presence of tetracycline residues in 20% (3/15) of beef muscle examined in South Africa; however, tetracycline residues were not found in the same samples by HPLC [27].

Oxytetracycline concentrations in normally slaughtered beef, emergency-slaughtered beef, and imported frozen beef samples examined ranged from 0.36 to 856, 0.45 to 986, and 0.69 to 930 µg/kg, with mean ± SE values of 245.5 ± 8, 378.6 ± 21, and 261.4 ± 16 µg/kg, respectively (Figure 1B). Significantly higher levels of oxytetracycline (*p* < 0.01) were detected in emergency-slaughtered beef than in normally slaughtered beef and imported frozen beef (Figure 1B).

A lower mean concentration of 148.17 µg/kg, with a range between 35.24 and 464.14 µg/kg, was recorded for oxytetracycline in sheep muscle marketed in Kuwait [11]. Lower findings were also observed in Zambia, where oxytetracycline concentrations in cattle meat ranged from 27.26 to 481.6 µg/kg, with a mean of 199.6 µg/kg [42]. Additionally, Bedada et al. [44] found lower mean oxytetracycline residue levels of 15.92, 64.85, and 108.35 µg/kg in beef samples from Debre Zeit, Nazareth, and Addis Ababa abattoirs in central Ethiopia, respectively. On the other hand, a much lower mean value of 48.6 µg/kg was detected for tetracycline in beef samples tested in South Africa [27]. Furthermore, Mingle et al. [41] reported that the mean concentrations of oxytetracycline in beef samples from Accra and Kumasi in Ghana were 7.6 and 5.7 µg/kg, respectively. The higher concentrations of tetracycline reported in the present study indicated the misuse and overuse of such an antibiotic in the treatment of farm animals, especially emergency-slaughtered ones.

### 2.5. Prevalence and Concentration of Sulphadimidine Residues (μg/kg) in Beef Samples

Sulphadimidine, also known as sulfamethazine, is a sulfonamide antibacterial that is commonly used in veterinary medicine, particularly for ruminants and poultry, to treat bacterial infections and as a growth promoter in animal feed due to its effectiveness and low cost, leading to a surge of sulfonamide-resistant bacterial species [17]. In the present study, sulphadimidine residues were detected in 41.7% (125/300) of the three categories of examined beef samples, while at the beef category level, sulphadimidine was detected in 32% (32/100), 63% (63/100), and 30% (30/100) of normally slaughtered beef, emergency-slaughtered beef, and imported frozen beef samples tested, respectively. Comparable findings were observed in Saudi Arabia, where sulphadimidine residues were detected in 36.52% and 28.22% of camel and sheep samples collected from Al-Ahsa central slaughterhouse; however, sulphadimidine residues were not detected in the examined cattle carcasses from the same abattoir [45]. A lower prevalence of 17.4% (39/224) was determined for sulfamethazine in beef samples examined in Zambia [42]. Furthermore, sulphanilamide was detected in only 6.6% (1/15) of beef samples analyzed in South Africa by HPLC [27].

The concentrations of sulphadimidine in normally slaughtered beef, emergency-slaughtered beef, and imported frozen beef samples examined ranged from 3.20 to 860, 0.23 to 1250, and 0.88 to 598 µg/kg, with mean ± SE values of 209.7 ± 6, 301.4 ± 15, and 115.7 ± 11 µg/kg, respectively (Figure 1C).

Emergency-slaughtered beef had significantly higher sulphadimidine levels (*p* < 0.01) than normally slaughtered beef and imported frozen beef, and the order of sulphadimidine concentration among the beef samples tested was: emergency-slaughtered beef > normally slaughtered beef > imported frozen beef (Figure 1C).

Higher concentrations of sulfamethazine were reported in samples collected from a local abattoir in Saudi Arabia, in a range of 3.64 and 4434.82 ng/g in camel samples and between 2.61 and 2554.98 ng/g in the sheep samples [45]. On the other hand, lower concentrations of sulfamethazine, ranging from 11.9 to 259.9 ng/g with a mean ± SD of 86.5 ± 75.9 ng/g, were detected in beef samples examined in Zambia [42] while a concentration of 65.3 µg/kg was determined in the single sulphanilamide-positive beef sample analyzed in South Africa by HPLC [27].

Variations in the prevalence and concentration of antibiotic residues observed between our findings and previous global studies can be attributed to a combination of regulatory, management, and analytical factors. Higher residue levels in locally slaughtered and emergency-slaughtered beef compared to imported beef reflect differences in the enforcement of withdrawal periods and farm-level veterinary oversight. In local production systems, emergency slaughter is frequently triggered by acute systemic infections, leading to slaughter before therapeutic agents are fully cleared from tissue [5,38]. Conversely, imported frozen beef originates from export-certified facilities bound by stringent international monitoring standards and mandatory holding periods [43]. Furthermore, numerical discrepancies across the published literature often stem from methodological variations; studies utilizing highly sensitive multi-residue HPLC or LC-MS/MS assays report higher detection rates near lower limits of detection (LOD) compared to studies employing less sensitive screening bioassays or ELISAs [10,24,27].

### 2.6. Acceptability of Beef Carcasses and Cuts Based on the Maximum Residue Limits (MRLs) of the Analyzed Antibiotic Residues

#### 2.6.1. Ciprofloxacin

**Figure 2:**
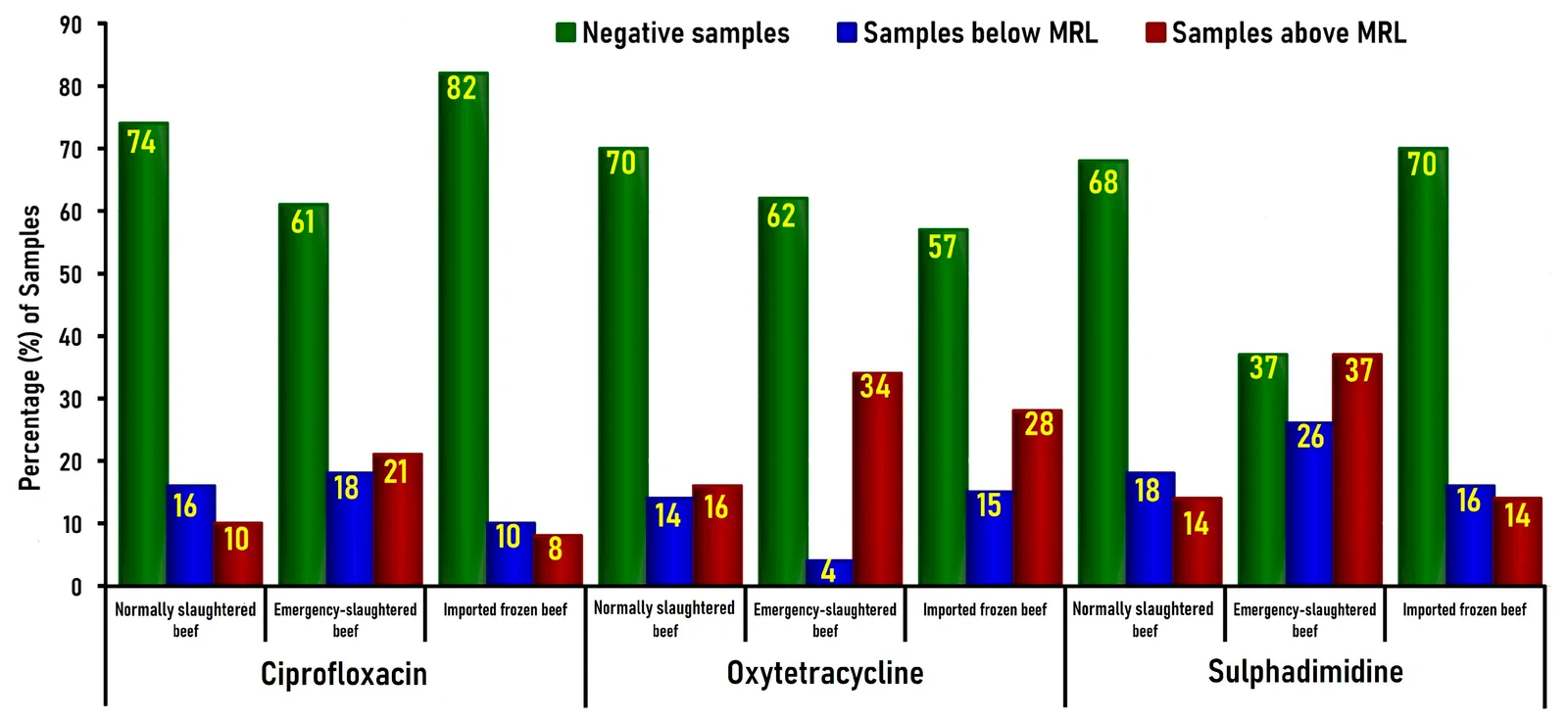
Percentage of negative, compliant (≤MRL), and non-compliant (>MRL) beef samples for ciprofloxacin, oxytetracycline, and sulphadimidine across normally slaughtered beef, emergency-slaughtered beef, and imported frozen beef categories (*n* = 100 per category), evaluated against European Union maximum residue limits (EU MRLs) for target antibiotics.

It has been revealed that 10% (10/100), 21% (21/100), and 8% (8/100) of normally slaughtered beef, emergency-slaughtered beef, and imported frozen beef samples, respectively, with an overall percentage of 13% (39/300) (Figure 2) exceeded the ciprofloxacin MRL of 100 µg/kg established by Regulation (EU) No. 37/2010, which applies to the combined concentration of enrofloxacin and ciprofloxacin in bovine muscle [21]. These samples were therefore considered non-compliant with the established regulatory requirements, highlighting the need for effective monitoring and control of fluoroquinolone residues in beef to ensure consumer safety. By comparison, Ramatla et al. [27] in South Africa detected ciprofloxacin residues in beef muscle at concentrations ranging from 89.6 to 146.1 µg/kg, with 33% (5/15) of the samples exceeding the recommended MRL of 100 µg/kg. Furthermore, 23.75% (19/80) of beef samples tested in Nigeria exceeded the MRL [39]. Conversely, ciprofloxacin concentrations in beef samples tested in Iran ranged from 0.1 to 43.2 µg/kg, and none of the samples exceeded the MRL [40].

#### 2.6.2. Oxytetracycline

In the present study, 26% (78/300) of the examined beef samples exceeded the MRL of 100 µg/kg set for oxytetracycline by the European Union [21], representing non-compliance rates of 16% (16/100) in normally slaughtered beef, 34% (34/100) in emergency-slaughtered beef, and 28% (28/100) in imported frozen beef. However, when evaluated against the higher MRL threshold of 200 µg/kg established for oxytetracycline by the Codex Alimentarius Commission [22], the overall non-compliance rate decreased to 18.3% (55/300), with corresponding rates of 14% (14/100), 26% (26/100), and 15% (15/100) in normally slaughtered beef, emergency-slaughtered beef, and imported frozen beef, respectively (Figure 2).

The residue levels observed in this study align with reports from other regions where oxytetracycline non-compliance remains significant. For instance, Sallam et al. [11] detected oxytetracycline concentrations in sheep muscle in Kuwait ranging from 35.24 to 464.14 µg/kg, with 45% (54/120) of positive samples exceeding MRLs. Similarly, in Zambia, oxytetracycline concentrations in cattle meat ranged from 27.26 to 481.6 µg/kg, with 45.5% (35/77) of positive samples exceeding the Codex MRL [42]. In central Ethiopia, Bedada et al. [44] reported oxytetracycline levels ranging from 3.0 to 429.3 µg/kg across abattoirs in Debre Zeit, Nazareth, and Addis Ababa, with non-compliance rates reaching 48.1% (51/106) and 48.3% (58/120) at the Nazareth and Addis Ababa abattoirs, respectively [44]. Conversely, complete compliance with EU limits has been documented in other studies; none of the beef samples (*n* = 15) analyzed in South Africa [27], Iran (*n* = 41) [40], or Ghana (*n* = 78) [41] exceeded the 100 µg/kg EU MRL threshold.

#### 2.6.3. Sulphadimidine

The present study revealed that 14% (14/100), 37% (37/100), and 14% (14/100) of normally slaughtered beef, emergency-slaughtered beef, and imported frozen beef samples tested in this study, respectively, with an overall percentage of 21.7% (65/300) exceeded the MRL of 100 µg/kg set for sulphadimidine by both the European Union [21] and Codex Alimentarius Commission [22], indicating non-compliance with the established regulatory standards. By comparison, 10% and 45% of sheep and camel muscles obtained from a local abattoir in Saudi Arabia exceeded the maximum permissible limits of sulfamethazine [45]. Moreover, 13 (33.3%) of the 39 sulphadimidine-positive beef samples analyzed in Zambia were above the MRL of 100 ng/g set for sulphadimidine by the EU standard [42]. Conversely, none of the sulfonamide-positive beef samples tested in South Africa exceeded the MRLs [27].

### 2.7. Health Risk Assessment of Antibiotic-Contaminated Beef Samples

The *EDI* of ciprofloxacin, oxytetracycline, and sulphadimidine was calculated for different age groups of the Egyptian population to characterize dietary exposure and evaluate the potential health hazards associated with consumption of beef containing these antibiotic residues. The resulting *EDI* values were compared with the corresponding acceptable daily intake (*ADI*) values to calculate the *HQ* (Table 1). The EDI range of ciprofloxacin for the different age groups of the Egyptian population in all beef samples examined ranged between 0.063 and 0.281 μg/kg bw, while the corresponding *HQ* values ranged from 0.032 to 0.141. For oxytetracycline, the *EDI* and *HQ* values ranged from 0.114 to 0.409 μg/kg bw and 0.0038 to 0.0136, respectively. For sulphadimidine, the *EDI* range was 0.053 to 0.325 μg/kg bw, while the corresponding *HQ* values ranged from 0.0011 to 0.0065 (Table 1).

In the absence of age-disaggregated national dietary survey data for Egypt, a uniform national per capita beef consumption rate of 13.4 kg/person/year (equivalent to 0.03671 kg/person/day) was used [46]. The mean body weights of Egyptian women and men aged 15–59 years were 77.1 and 79.1 kg, respectively, according to the Egypt Health Issues Survey [47]. For schoolchildren aged 5–14 years, an average body weight of 34 kg was adopted based on Egyptian growth charts [48]. The *EDI* of each antibiotic residue was higher in schoolchildren (5–14 years; 34 kg) than in women (15–59 years; 77.1 kg) and men (15–59 years; 79.1 kg). This finding reflects the higher exposure per unit body weight among younger consumers and indicates that children may have greater dietary exposure to antibiotic residues on a body-weight basis than adults (Table 1).

The highest *HQ* values were observed in schoolchildren (5–14 years; 34 kg) consuming emergency-slaughtered beef, reflecting their increased vulnerability due to lower body weight. The maximum *HQ* values were 0.141, 0.0136, and 0.0065 for ciprofloxacin, oxytetracycline, and sulphadimidine, respectively; even so, these values are still 7.1, 73.53, and 153.85 times lower than the threshold of 1, respectively (Table 1). The *HQ* values are higher for ciprofloxacin compared to oxytetracycline and sulphadimidine (Table 1). Overall, the current findings revealed that there is no significant health risk for schoolchildren and adults from the consumption of beef (Table 1).

Frequent exposure to antibiotic residues in food can cause several adverse effects on human health, including allergies, digestive disorders, anaphylactic shocks, mutagenicity, carcinogenicity, teratogenicity, and neurological disorders. Furthermore, it may result in therapeutic failure, particularly in children, and the development of antibiotic-resistant bacteria [11,20]. The *EDI* was calculated using the equation established by Chandrakar et al. [37], Juan et al. [35], and Rahman et al. [26]. The average daily red meat consumption in Egypt was estimated at 36.7 g/day [46] while the assumed body weights were 34 kg for school-aged children (5–14 years), 80.7 kg for women, and 81.3 kg for men aged 15–59 years (Table 1). The Acceptable Daily Intake (*ADI*) is the estimated amount of antibiotic residue that can be safely ingested daily over a lifetime without significant health risks, expressed on a body weight basis. The *ADI* of ciprofloxacin is 2 µg/kg bw per day [36] while the *ADIs* for oxytetracycline and sulphadimidine are 30 and 50 μg/kg bw per day, respectively [22].

Interestingly, emergency-slaughtered beef exhibited the highest *EDI* for each antibiotic residue tested across all age groups compared to normally slaughtered beef and imported frozen ones (Table 1), indicating non-compliance with withdrawal periods of drugs before slaughter. Moreover, imported frozen beef also showed relatively higher *EDI* values than normally slaughtered beef for ciprofloxacin and oxytetracycline, but it had relatively lower *EDI* values compared to normally slaughtered beef for sulphadimidine.

On the other hand, all *HQ* values were below 1, indicating that the estimated dietary exposure to the investigated antibiotic residues through consumption of the examined beef samples was below the levels expected to cause significant health hazards. The highest *HQ* values were recorded for ciprofloxacin, although the maximum value (0.141) remained well below the threshold of 1. This suggests a lower potential health risk from dietary exposure to the examined antibiotic residues under the consumption assumptions used in the present study [37,38].

To date, only limited data are available concerning the health risk assessment of dietary exposure to antibiotic residues through food of animal origin. Similar findings were mentioned by Sallam et al. [11], who found *HQ* values for oxytetracycline and tetracycline combination in sheep muscle, liver, and kidney samples tested in Kuwait were 153 times lower than the levels expected to cause health hazards. Furthermore, Haque et al. [20] found that the *HQ* values for ciprofloxacin, tetracycline, and oxytetracycline residues in all chicken samples examined were below 1, suggesting low-risk exposure for human health. Likewise, Chandrakar et al. [37] estimated that the *HQ* values for dietary exposure to ciprofloxacin, tetracycline, oxytetracycline, and sulfamethazine through consumption of chicken meat were below 1, indicating a negligible health risk for adults and children. Moreover, exposure to antibiotics, even at low doses, could result in the development of antibiotic-resistant bacteria, resulting in long-term health consequences for humans and may cause therapeutic failure, particularly in children. Consequently, strict adherence to withdrawal periods for veterinary drugs is crucial to minimize antibiotic residues in meat samples and protect public health.

## 3. Materials and Methods

### 3.1. Beef Tissue Sampling

A total of 300 beef samples were collected from five major administrative districts of Ismailia Governorate, Egypt (Ismailia City, Fayed, El-Qantara West, El-Qantara East, and El-Tell El-Kebir), between January and September 2023. The sample size was equally allocated among three predefined beef categories (*n* = 100 per category): beef from normally slaughtered cattle, beef from emergency-slaughtered cattle, and commercially marketed imported frozen beef of Brazilian origin. This balanced allocation was adopted to facilitate comparative assessment of antibiotic residues among the three beef categories. Samples were distributed across the five districts as follows: Ismailia City (80 samples), Fayed (60 samples), El-Qantara West (50 samples), El-Qantara East (40 samples), and El-Tell El-Kebir (70 samples). Samples of normally slaughtered beef were collected from five licensed slaughterhouses/retail outlets distributed across the study districts, whereas samples from emergency-slaughtered cattle were obtained from eight farms/slaughtering sites. Imported frozen beef samples were collected from six retail outlets/supermarkets. Sampling sites and retail outlets were purposively selected from licensed and operating establishments to ensure geographic coverage of the five administrative districts and availability of the three predefined beef categories during the study period.

Approximately 100 g of ribeye muscle (*Longissimus dorsi*) was aseptically collected from each individual beef sample using sterile instruments and placed in sterile, appropriately labelled containers. For normally slaughtered and emergency-slaughtered cattle, the ribeye cut was obtained directly from the carcass or slaughtered animal under veterinary supervision, where applicable. For imported frozen beef, individual commercially packaged units were obtained from the selected retail outlets, and approximately 100 g of ribeye (Longissimus dorsi) muscle was collected from each sampled unit after thawing under controlled conditions. Samples were transported to the laboratory under appropriate cold-chain conditions and stored at −20 °C [23] for approximately 1–3 months until preparation and HPLC analysis for determination of the targeted antibiotic residues.

**Figure 3:**
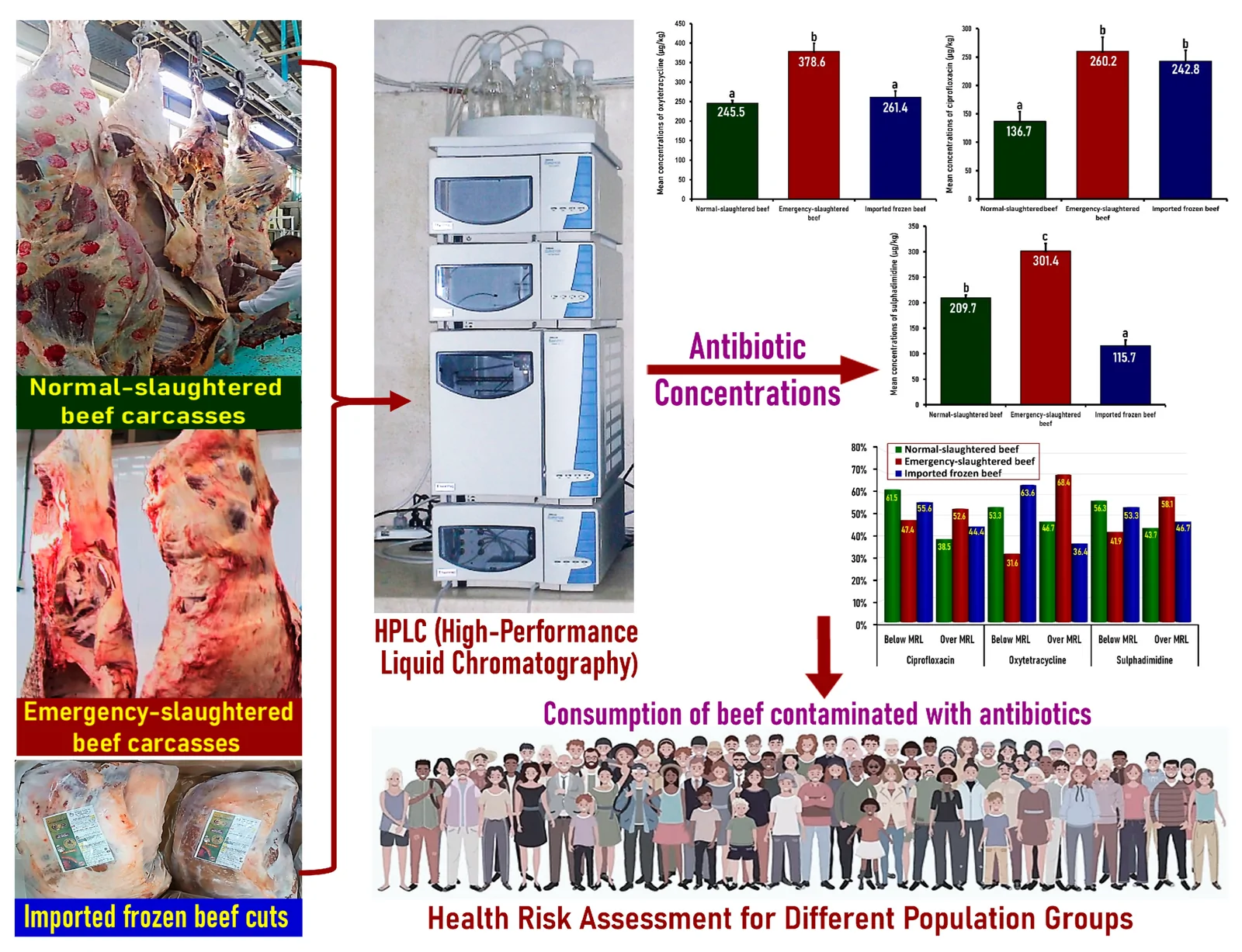
The schematic overview of the experimental design and key findings. Beef samples from normally slaughtered cattle, emergency-slaughtered cattle, and imported frozen beef were subjected to HPLC-based determination of oxytetracycline, ciprofloxacin, and sulfadimidine residues. Residue concentrations and compliance with maximum residue limits (MRLs) were assessed and compared among sample categories. The generated residue data were subsequently used to estimate dietary exposure and characterize potential health risks for different consumer population groups.

This study did not involve live animals or human participants. Beef samples were collected post-slaughter from licensed slaughterhouses and retail markets as part of routine food safety monitoring. Therefore, ethical approval was not required in accordance with institutional and national guidelines for research involving animal-derived food products. The schematic overview of the experimental design and key findings of the present study is shown in Figure 3.

### 3.2. Chemicals and Reagents

High-purity analytical standards were used in the current study; ciprofloxacin, oxytetracycline, and sulphadimidine standards were obtained from Sigma-Aldrich (Merck KGaA, Darmstadt, Germany). HPLC-grade methanol, acetonitrile, and water were acquired from POCH SA (Avantor Performance Materials Poland S.A., Gliwice, Poland). Formic acid (85%) was purchased from El-Nasr Pharmaceutical Chemicals Company, Abu Zaabal, Egypt. Potassium dihydrogen phosphate, citric acid, trichloroacetic acid, and nitric acid (30%) were obtained from Sigma-Aldrich (Merck KGaA, Darmstadt, Germany).

### 3.3. Equipment and Instruments

The chromatographic analysis was conducted using an HPLC system equipped with a constant-flow liquid chromatography pump (Surveyor, Thermo Scientific, Waltham, MA, USA) to supply the solvent mixture to the analytical column. Sample injection was conducted using an autosampler (Surveyor, Thermo Scientific, USA), and detection was carried out with a variable-wavelength PDA (Photodiode Array) detector (Surveyor, Thermo Scientific, USA). The data analysis was performed automatically using ChromoQuest 4.2 software (Surveyor, Thermo Scientific, USA). Separation was performed by a Hypersil Gold C18 column (150 × 4.6 mm, particle size 5 µm, Surveyor, Thermo Scientific, USA). MCE membrane syringe filters with 0.45 µm pore size were used to ensure the purity of the sample (Carrigtwohill, Cork, Ireland). The ultrasonic bath (Model 3510, Branson, Danbury, CT, USA) was used to facilitate proper homogenization of the samples.

### 3.4. Quantitative Analysis of Antibiotics by High-Performance Liquid Chromatography (HPLC)

The validation parameters of the antibiotic analysis by HPLC, including calibration curve range, retention time, regression equation, correlation coefficient, slope, limit of detection (LOD), limit of quantification (LOQ), recovery, intraday and interday accuracy, and precision, are presented in the Supplementary Materials File (Table S1). Calibration curves exhibited excellent linearity with correlation coefficients (R^2^) of 0.9999 for ciprofloxacin, 0.9996 for oxytetracycline, and 0.9997 for sulphadimidine.

#### 3.4.1. Chromatographic Equipment and Preparation of Standards 

Chromatographic separation for all target residues was performed at 25 °C using a Hypersil Gold C18 column (150 × 4.6 mm, 5 μm) and an injection volume of 20 μL. Peaks were integrated and residues quantified according to peak areas using ChromoQuest 4.2 software. Stock solutions (1000 μg/mL) were prepared individually and serially diluted to obtain working standards in both the mobile phase eluent and spiked beef matrix.

#### 3.4.2. Quantitative Determination of Ciprofloxacin

Ciprofloxacin was extracted from beef tissue according to Verdon et al. [49]. Frozen samples were thawed, trimmed of external fat and fascia, finely ground, and homogenized. A 2-g portion was mixed with 8 mL of 5% trichloroacetic acid, vortexed for 1 min, shaken for 10 min, and centrifuged at 14,000 rpm for 5 min at 4 °C. The supernatant was filtered through a 0.45 μm nylon membrane filter prior to HPLC injection. The mobile phase consisted of acetonitrile and 0.01 M phosphoric acid buffer (pH 3.0) (25:75, *v*/*v*) at a flow rate of 1.0 mL/min. Photodiode array (PDA) detection was performed at 278 nm, with a retention time of 6.36 min [50]. Calibration standards were prepared at 0.05, 0.1, 0.25, 0.5, and 1 μg/mL; the stock solution was prepared in acidified water (0.1% formic acid).

#### 3.4.3. Quantitative Determination of Oxytetracycline

Oxytetracycline was extracted according to Senyuva et al. [51]. Following thawing, trimming, and grinding, 2 g of beef tissue was mixed with 0.1 g citric acid, 1 mL nitric acid (30%), 4 mL methanol, and 1 mL deionized water. The mixture was vortexed, sonicated for 15 min, and centrifuged at 5300 rpm for 10 min. The supernatant was filtered through a 0.45 μm nylon membrane filter before HPLC injection. The mobile phase consisted of acetonitrile and 0.1% formic acid at a flow rate of 1.0 mL/min. PDA detection was performed at 350 nm, with a retention time of 6.06 min [17]. Calibration standards were prepared at 0.1, 0.5, 1, 5, and 10 μg/mL; the stock solution was prepared in methanol–water (50:50, *v*/*v*) containing 0.01 M oxalic acid.

#### 3.4.4. Quantitative Determination of Sulphadimidine

Sulphadimidine was extracted according to Ma et al. [52]. Briefly, 2 g of finely ground beef tissue was extracted with 5 mL of acetonitrile containing 0.2% formic acid. The mixture was vortexed for 1 min, sonicated for 30 min, and centrifuged at 8000 rpm for 5 min. The supernatant was subjected to solid-phase extraction (SPE) using cartridges conditioned with 1000 μL each of water and methanol. After loading, the extract was eluted using 1000 μL of water at a flow rate of 1000 μL/min. The mobile phase consisted of 0.01 M potassium dihydrogen phosphate buffer and methanol (70:30, *v*/*v*) at a flow rate of 1.0 mL/min. PDA detection was performed at 264 nm, with a retention time of 4.85 min [52]. Calibration standards were prepared at 0.1, 0.5, 1, 5, and 10 μg/mL; the stock solution was prepared in methanol–water (50:50, *v*/*v*).

### 3.5. Assessment of Health Risks Associated with the Consumption of Beef Samples Tested

#### 3.5.1. Estimated Daily Intake (*EDI*)

The *EDI* of antibiotic agents analyzed, comprising ciprofloxacin, oxytetracycline, and sulphadimidine, was calculated to assess health risks associated with the consumption of normally slaughtered beef, emergency-slaughtered beef, and imported frozen beef following the equation set by Chandrakar et al. [37], Juan et al. [35], and Rahman et al. [26].

*EDI* = (μg/kg bw) = *MC* × *DC*/*BW*.

*MC* (μg/kg) is the mean concentration of antibiotic residues. *DC* is the daily red meat consumption rate in Egypt, estimated by the Ministry of Agriculture and Land Reclamation at an average of 13.4 kg per capita/year, which is equivalent to 36.7 g/day (0.0367 kg/day) [46]. *BW* is the average body weight of Egyptian consumers, which is estimated according to the Egypt Health Issues Survey at 77.1 kg for women and 79.1 kg for men aged 15–59 years [47], and at 34 kg for schoolchildren aged 5–14 years, adopted based on Egyptian growth charts [48].

#### 3.5.2. Hazard Quotient (*HQ*)

The *HQ* was estimated after calculating the *EDI* using the formula set by Pogurschi et al. [38] and Rahman et al. [26].

*HQ* = *EDI*/*ADI*

*ADI* (Acceptable Daily Intake) is the daily intake of antibiotic residue allowed for consumption over a lifetime without an appreciable health risk. According to the Joint FAO/WHO Expert Committee on Food Additives, WHO Technical Report Series, No. 879 [36], the *ADI* of ciprofloxacin is 2 µg/kg bw per day, while the *ADIs* for oxytetracycline and sulphadimidine are 30 and 50 μg/kg bw per day, respectively [22]. An *HQ* of <1.0 indicates acceptable and negligible risk, which does not pose any significant health risk, while an *HQ* > 1.0 suggests an unacceptable risk that could cause potential adverse effects on human health [37,38].

### 3.6. Statistical Analysis

The estimation of antibiotic residues in the beef samples examined was carried out in triplicate measurements. Data were organized using Microsoft Excel and analyzed using IBM SPSS Statistics for Windows (Version 28.0; IBM Corp., Armonk, NY, USA). Descriptive statistics were expressed as mean ± standard error (SE), median, and range. The suitability of parametric statistical procedures was evaluated by verifying data normality using the Shapiro–Wilk test and assessing the homogeneity of variances using Levene’s test. For residue concentration data exhibiting right-skewness, values were log_10-transformed prior to hypothesis testing to satisfy parametric assumptions.

The use of parametric one-way Analysis of Variance (ANOVA) followed by Tukey’s honest significant difference (HSD) post hoc test was justified due to the large and balanced sample size (*n* = 100 per group, total *N* = 300), invoking the Central Limit Theorem to ensure normality of the sampling distribution. Differences in residue prevalence rates among beef groups were evaluated using Pearson’s Chi-square (χ^2^) test. All statistical tests were two-tailed, and differences in antibiotic concentrations among the different beef categories tested were considered statistically significant at *p* < 0.05 or *p* < 0.01.

## 4. Conclusions

The current study demonstrated widespread contamination of normally slaughtered, emergency-slaughtered, and imported frozen beef samples with ciprofloxacin, oxytetracycline, and sulphadimidine, with a notable proportion of samples exceeding established MRLs. Residue concentrations and non-compliance rates were highest in emergency-slaughtered beef, underscoring potential failure to observe withdrawal periods prior to emergency slaughter. Dietary health risk assessment indicated that while exposure levels yielded *HQ* values below the threshold of safety concern across all evaluated demographic groups, schoolchildren exhibited higher relative exposure levels than adults due to lower body weight. Despite the absence of immediate non-carcinogenic health risks, chronic exposure to low residue levels presents broader public health concerns regarding the selection and proliferation of antibiotic-resistant bacteria. Consequently, routine residue surveillance in animal-derived foods, rational veterinary drug use, and strict enforcement of withdrawal periods remain essential to ensure meat safety and protect public health.

## Figures and Tables

**Table 1:** Health risk assessment of ciprofloxacin, oxytetracycline, and sulphadimidine residues in normally slaughtered beef, emergency-slaughtered beef, and imported frozen beef.

Antimicrobial Agents	Sample Source	*EDI* (μg/kg bw) for the Different Age Groups of the Egyptian Population			*ADI* (μg/kg bw)	* *HQ* for the Different Age Groups of the Egyptian Population		
		Schoolchildren (5–14 Years; 34 kg bw)	Women (15–59 Years; 77.1 kg bw)	Men (15–59 Years; 79.1 kg bw)		Schoolchildren (5–14 Years; 34 kg bw)	Women (15–59 Years; 77.1 kg bw)	Men (15–59 Years; 79.1 kg bw)
Ciprofloxacin	Normally slaughtered beef	0.148	0.065	0.063	2 ^a^	0.074	0.033	0.032
	Emergency-slaughtered beef	0.281	0.124	0.121		0.141	0.062	0.061
	Imported frozen beef	0.262	0.116	0.113		0.131	0.058	0.057
Oxytetracycline	Normally slaughtered beef	0.265	0.117	0.114	30 ^b^	0.0088	0.0039	0.0038
	Emergency-slaughtered beef	0.409	0.180	0.176		0.0136	0.006	0.0058
	Imported frozen beef	0.282	0.124	0.121		0.0094	0.0041	0.0040
Sulphadimidine	Normally slaughtered beef	0.226	0.099	0.097	50 ^b^	0.0053	0.0019	0.0019
	Emergency-slaughtered beef	0.325	0.143	0.139		0.0065	0.0028	0.0027
	Imported frozen beef	0.125	0.055	0.053		0.0025	0.0011	0.0011

^a^ JECFA/WHO Report 879 [36]; ^b^ Codex Alimentarius Commission [22]. * *HQ* ≤ 1.0 indicates no appreciable health risk; *HQ* > 1 indicates potential human health risk.

## Data Availability

All data generated or analyzed during this study are included in this published article.

## Supplementary Materials

The following supporting information can be downloaded at: https://www.mdpi.com/article/10.3390/antibiotics15090932/s1, Figure S1. HPLC standard calibration curves for (A): ciprofloxacin (0.05, 0.1, 0.25, 0.5, and 1 μg/mL), (B): Oxytetracycline (0.1, 0.5, 1, 5, and 10 μg/mL), and (C): Sulphadimidine (0.1, 0.5, 1, 5, and 10 μg/mL). Chromatogram of the blank sample (D). Figure S2. Chromatograms of the stand-ards and extracted beef samples. Liquid chromatograms of 1 μg/mL ciprofloxacin standard (A), oxytetracycline standard (B), and sulphadimidine standard (C), and the corresponding extracts from beef samples: ciprofloxacin (D), oxytetracycline (E), and sulphadimidine (F). Table S1. Validation sheet of the HPLC analytical method.
