# Supplementary material for: Health Risk Assessment of Antibiotic Residues in Normally Slaughtered, Emergency-Slaughtered, and Imported Beef in Egypt
Source: Antibiotics (Basel). 2026 Sep 20;15(9):932. doi: 10.3390/antibiotics15090932 (PMC13603705; doi:10.3390/antibiotics15090932)
Supplement: Supplementary file 1 [file antibiotics-15-00932-s001.zip › antibiotics-4546443-supplementary.pdf]

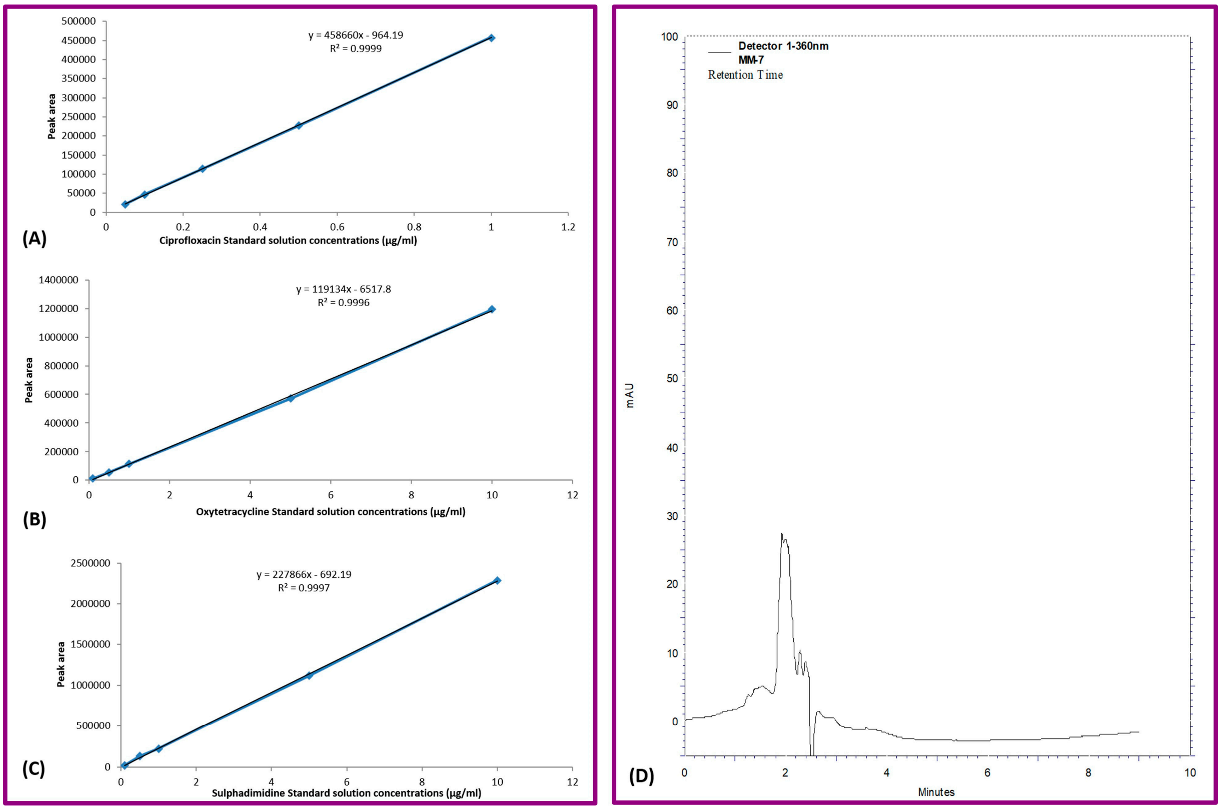

Figure S1. HPLC standard calibration curves for (A): ciprofloxacin (0.05, 0.1, 0.25, 0.5, and 1 µg/mL), (B): Oxytetracycline (0.1, 0.5, 1, 5, and 10 µg/mL), and (C): Sulphadimidine (0.1, 0.5, 1, 5, and 10 µg/mL). Chromatogram of the blank sample (D).

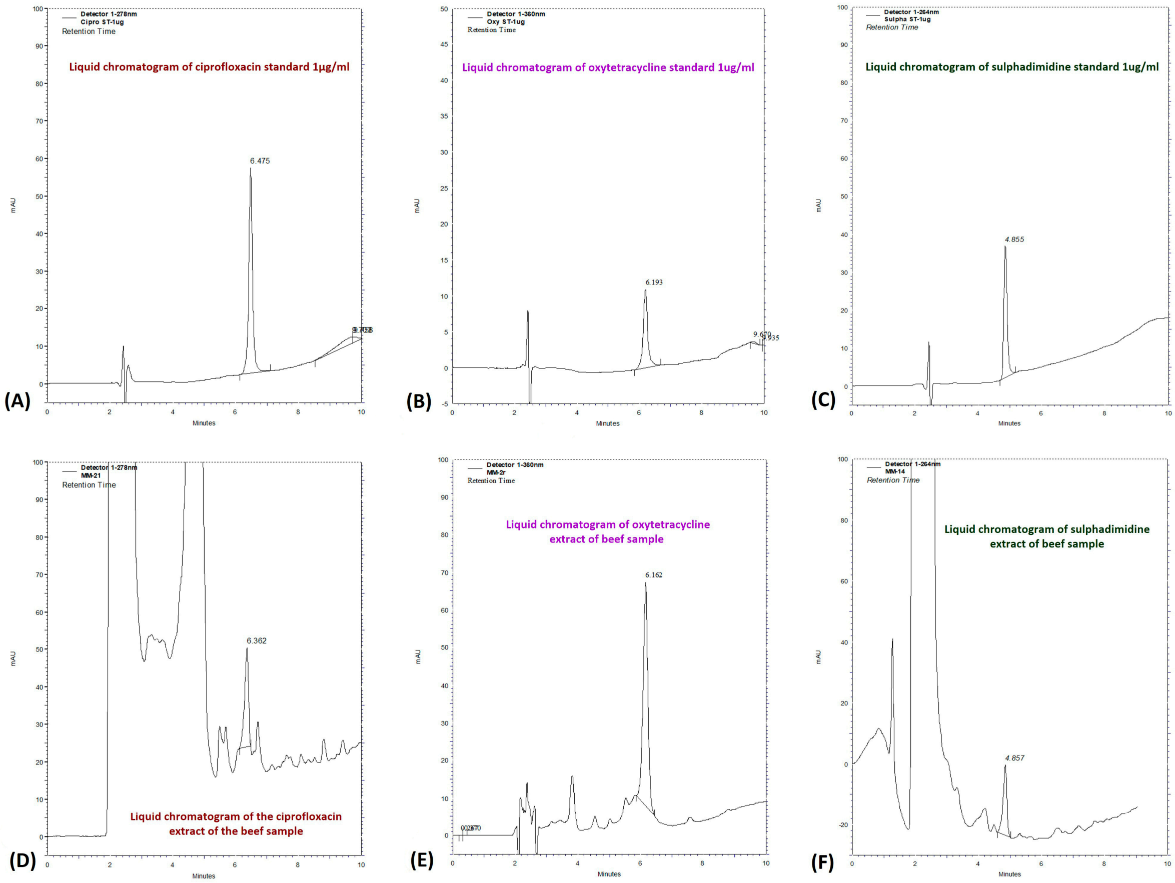

Figure S2. Chromatograms of the standards and extracted beef samples. Liquid chromatograms of 1 µg/mL ciprofloxacin standard (A), oxytetracycline standard (B), and sulphadimidine standard (C), and the corresponding extracts from beef samples: ciprofloxacin (D), oxytetracycline (E), and sulphadimidine (F).

Table S1. Validation sheet of the HPLC analytical method.

Parameter	Ciprofloxacin	Oxytetracycline	Sulphadimidine
**Calibration curve range (μg/ml)**	0.05,0.1, 0.25, 0.5, 1	0.1, 0.5, 1, 5,10	0.1, 0.5, 1, 5,10
**Retention time (min)**	6.36 ± 0.03	6.06 ± 0.04	4.85 ± 0.02
**Regression equation**	y = 458660x - 964.19	y = 119134x - 6517.8	y = 227866x - 692.19
**Correlation coefficient**	0.9999	0.9996	0.9997
**Slope (a)**	458660	119134	227866
**Intercept (b)**	964.19	6517.8	692.19
**Limit of detection LOD (μg/g)**	0.002	0.015	0.003
**Limit of Quantification (μg/g)**	0.005	0.05	0.01
**Recovery**	92-98	96-99	92-96
**Intraday precision (RSD%)**	0.06	0.22	0.083
**Interday precision (RSD%)**	0.07	0.47	0.11
